# Angina: contemporary diagnosis and management

**DOI:** 10.1136/heartjnl-2018-314661

**Published:** 2020-02-12

**Authors:** Thomas Joseph Ford, Colin Berry

**Affiliations:** 1 BHF Cardiovascular Research Centre, University of Glasgow College of Medical Veterinary and Life Sciences, Glasgow, UK; 2 Department of Cardiology, Gosford Hospital, Gosford, New South Wales, Australia; 3 Faculty of Health and Medicine, The University of Newcastle, Newcastle, NSW, Australia

**Keywords:** cardiac catheterization and angiography, chronic coronary disease, percutaneous coronary intervention, coronary artery disease

**Learning objectives**

- Around one half of angina patients have no obstructive coronary disease; many of these patients have microvascular and/or vasospastic angina.
- Tests of coronary artery function empower clinicians to make a correct diagnosis (rule-in/rule-out), complementing coronary angiography.
- Physician and patient education, lifestyle, medications and revascularisation are key aspects of management.

## Introduction

Ischaemic heart disease (IHD) remains the leading global cause of death and lost life years in adults, notably in younger (<55 years) women.^1^ Angina pectoris (derived from the Latin verb ‘angere’ to strangle) is chest discomfort of cardiac origin. It is a common clinical manifestation of IHD with an estimated prevalence of 3%–4% in UK adults. There are over 250 000 invasive coronary angiograms performed each year with over 20 000 new cases of angina. The healthcare resource utilisation is appreciable with over 110 000 inpatient episodes each year leading to substantial associated morbidity.^2^ In 1809, Allen Burns (Lecturer in Anatomy, University of Glasgow) developed the thesis that myocardial ischaemia (supply:demand mismatch) could explain angina, this being first identified by William Heberden in 1768. Subsequent to Heberden’s report, coronary artery disease (CAD) was implicated in pathology and clinical case studies undertaken by John Hunter, John Fothergill, Edward Jenner and Caleb Hiller Parry.^3^ Typically, angina involves a relative deficiency of myocardial oxygen supply (ie, ischaemia) and typically occurs after activity or physiological stress (box 1).

Box 1 **Definition of angina (NICE guidelines)^32^**

Typical angina: (requires all three)

- Constricting discomfort in the front of the chest or in the neck, shoulders, jaw or arms.
- Precipitated by physical exertion.
- Relieved by rest or sublingual glyceryl trinitrate within about 5 min
  - Presence of two of the features is defined as atypical angina.
  - Presence of one or none of the features is defined as non-anginal chest pain.
  - Stable angina may be excluded if pain is non-anginal *provided* clinical suspicion is not raised based on other aspects of the history and risk factors.
  - Do not define typical, atypical and non-anginal chest pain differently in men and women or different ethnic groups.

**Figure 1:**
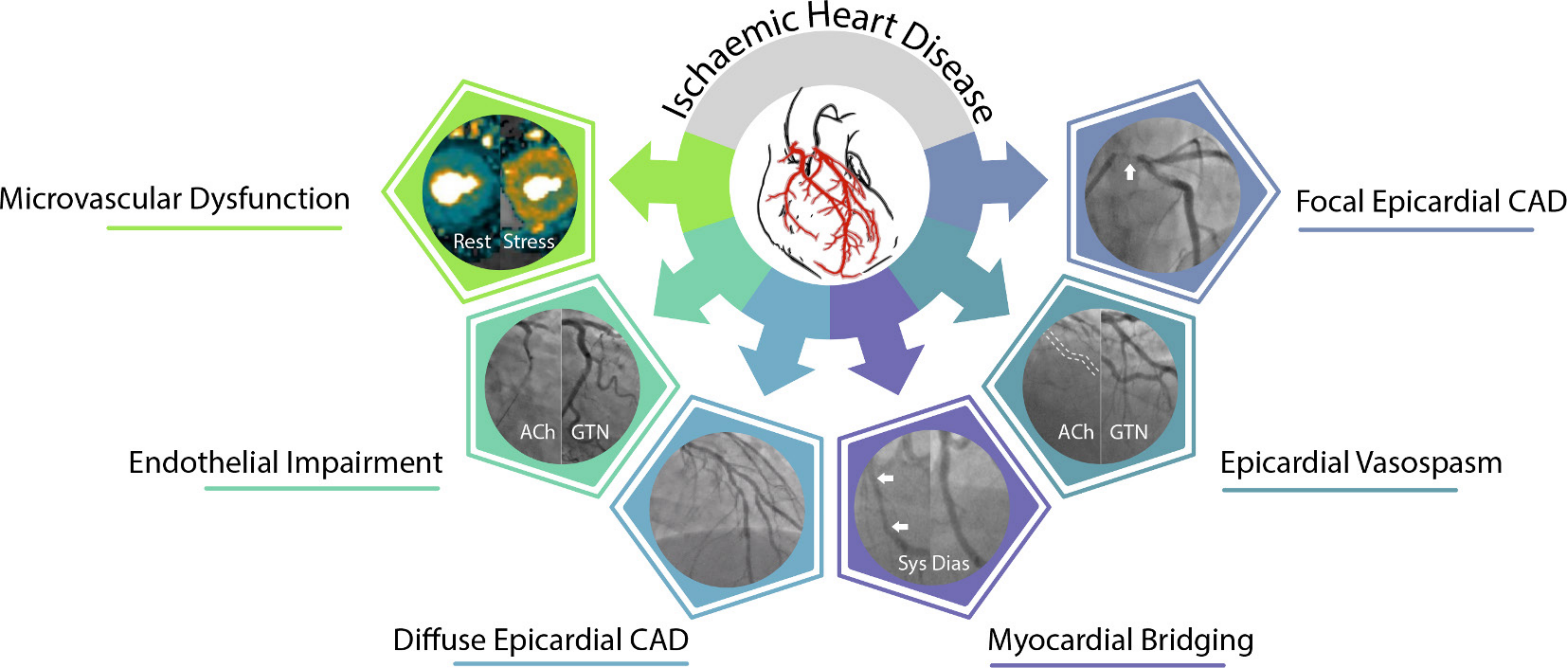
Reappraisal of ischaemic heart disease pathophysiology. Distinct functional and structural mechanisms can affect coronary vascular function and frequently coexist leading to myocardial ischaemia. CAD, coronary artery disease.

Six decades have passed since the first reported invasive coronary angiogram; however, many physicians still consider detecting obstructive epicardial CAD on coronary angiography a *‘sine qua non’* for the diagnosis of angina.^4^ The detection of obstructive CAD allows evidence-based medical treatment and consideration of myocardial revascularisation. However, underlying pathophysiology is more nuanced with contributions from anatomical atherosclerotic and/or functional alterations of epicardial vessels and/or microcirculation (figure 1).^5^ ESC guidelines^6^ have revised nomenclature (‘Chronic Coronary Syndromes’) in part reflecting the importance of patients with signs and symptoms of ischaemia without obstructive coronary artery disease—INOCA.^7 8^ Around half of all patients with angina undergoing elective coronary angiography have no obstructive epicardial CAD.^9^ This large, heterogeneous chronic coronary syndrome is comprised of distinct vasomotor disorders including microvascular angina (MVA) and/or vasospastic angina (VSA)—the two most common underlying disorders of coronary vascular function in the INOCA population. Crucially, we stress that there are often multiple mechanisms of myocardial ischaemia occurring in various coronary compartments via different mechanisms. These frequently coexist in combination; however, an appreciation of this fact can help stratify treatment and help us understand patients with poor treatment response (eg, angina after revascularisation).

We begin by classifying angina according to pathophysiology. We then consider the current guidelines and their strengths and limitations for assessing patients with recent onset of stable chest pain. We review non-invasive and invasive functional tests of the coronary circulation with linked management strategies. Finally, we point to future directions providing hope for improved patient outcomes and development of targeted disease-modifying therapy. The aim of this educational review is to provide a contemporary approach to diagnosis and management of angina taking into consideration epicardial coronary disease, microcirculatory dysfunction and coronary vasospasm.

## Contemporary angina classification by pathophysiology

The clinical history is of paramount importance to initially establish whether the nature of the presenting symptoms is consistent with angina (box 1). Indeed, recent data supports specialist physicians under-recognise angina in up to half of their patients.^10^ Furthermore, contemporary clinical trials of revascularisation in stable IHD including the ISCHEMIA trial highlight the importance of good clinical history and listening to our patients to determine the nature and frequency of symptoms which helps to plan management. We propose a classification of angina that aligns with underlying aetiology and related management (table 1).

**Table 1:** Classification of angina by pathophysiology

Angina with obstructive CAD		
Obstructive CAD	Flow limiting epicardial coronary artery disease*	>90% stenosis in a major coronary vessel Fractional flow reserve ≤0.80 or NHPR<0.90 Intermediate coronary stenosis in single major vessel with documented ischaemia
**Symptoms and/or signs of ischaemia but no obstructive CAD (INOCA)**		
Microvascular angina†^17^	*1. Symptoms of myocardial ischaemia*	Effort and/or rest angina Angina equivalents (ie, shortness of breath)
	2. Absence of obstructive CAD*	FFR>0.80 NHPR>0.89 Absence of flush ostial branch vessel occlusions
	*3. Objective evidence of myocardial ischaemia*	Ischaemic ECG changes during an episode of chest pain Stress-induced chest pain and/or ischaemic ECG changes in the presence or absence of transient/reversible abnormal myocardial perfusion and/or wall motion abnormality
	*4. Coronary microvascular dysfunction*	Impaired CFR (≤2.0 or≤2.5 depending on methods used) Increased coronary microvascular resistance (eg, IMR>25, HMR≥2.5 mm Hg cm^–1^ s) Coronary microvascular spasm, defined as reproduction of symptoms, ischaemic ECG shifts but no epicardial spasm during acetylcholine testing. Coronary slow flow phenomenon, defined as TIMI frame count >25
Vasospastic angina‡^42^	*Nitrate-responsive angina*	At least one of: Rest angina—especially between night and early morning Marked diurnal variation in exercise tolerance—reduced in morning Precipitated by hyperventilation Calcium channel blockers (but not β-blockers) suppress episodes
	*Ischaemic ECG changes*	*During spontaneous episode, any one of the following in at least two contiguous leads:* ST segment elevation ≥0.1 mV ST segment depression ≥0.1 mV New negative U waves
	*Coronary artery spasm*	*Either spontaneously or in response to provocation (eg, acetylcholine):* Transient total or subtotal coronary artery occlusion (>90% constriction) Reproduction of angina symptoms Ischaemic ECG changes

*The finding of obstructive epicardial disease does not exclude other important contributors to ischaemia (microvascular dysfunction and/or vasospasm). The physiological ischaemic lesion thresholds are drawn from 2018 ESC guidelines for myocardial revascularisation and randomised trials; however, the authors acknowledge that majority of lesions with grey-zone physiology values (eg, FFR 0.75–0.82) are not associated with downstream myocardial ischaemia (NCT02425969—Dr B Hennigan, Personal Correspondence).

†Definitive MVA is only diagnosed if all four criteria are present. Suspected MVA is diagnosed if criteria 1 and 2 are met but only one of the final two criteria are met (either objective evidence of ischaemia (criterion 3) or evidence of coronary microvascular dysfunction (criterion 4).

‡'Definitive vasospastic angina’ is diagnosed if nitrate-responsive angina is evident during spontaneous episodes and either the transient ischaemic ECG changes during the spontaneous episodes or coronary artery spasm criteria are fulfilled. ‘Suspected vasospastic angina’ is diagnosed if nitrate-responsive angina is evident during spontaneous episodes but transient ischaemic ECG changes are equivocal or unavailable and coronary artery spasm criteria are equivocal. NHPR (eg, iwFR, dPR).

CAD, coronary artery disease; CFR, coronary flow reserve; CFR, coronary flow reserve; dPR, diastolic pressure ratio; FFR, fractional flow reserve; HMR, hyperaemic microvascular resistance; IMR, index of microcirculatory resistance; MVA, microvascular angina; NHPR, non-hyperaemic pressure ratio.

### Angina with obstructive coronary artery disease

2018 ESC guidelines on myocardial revascularisation define obstructive CAD as coronary stenosis with documented ischaemia, a haemodynamically relevant lesion (ie, fractional flow reserve (FFR) ≤0.80 or non-hyperaemic pressure ratio (NHPR) (eg, iwFR≤0.89)) or >90% stenosis in a major coronary vessel (table 1). There is renewed interest in NHPRs (iwFR, resting full-cycle ratio (RFR) and diastolic pressure ratio (dPR)) as data have emerged in support of numerical equivalency between these indices suggesting all can be used to guide treatment strategy.^11^ Angina with underlying obstructive CAD allows symptom guided myocardial revascularisation (often with percutaneous coronary intervention (PCI)) and is effective in reducing ischaemic burden and symptoms (in many patients). Recent studies have served evidence that functional coronary disorders overlap and may contribute to angina even in patients with obstructive epicardial CAD. Dynamic changes in lesion or vessel ‘tone’ and propensity to vasoconstriction is important and may cause rest angina that is frequently overlooked in patients with obstructive CAD.^12^ During invasive physiological assessment of ischaemia during exercise, Asrress *et al* showed that ischaemia developed at FFR averaging≈0.76 which is not often observed with adenosine induced hyperaemia.^13^ This finding implies there are other important drivers of subendocardial ischaemia (myocardial supply:demand factors). Furthermore, it reinforces that angina is not synonymous with ischaemia or flow-limiting coronary disease (eg, abnormal FFR or NHPR). Coronary anatomy and physiology should not be considered in isolation but in the context of the patient.

#### Angina-myocardial ischaemia discordance

Although obstructive CAD or microvascular dysfunction may be present, the link between ischaemia and angina is not clearcut. The ‘ischaemic threshold’ (the heart rate-blood pressure product at the onset of angina) has intraindividual and interindividual variability.^14^ Innate differences in vascular tone and endocrine changes (eg, menopause) may influence propensity to vasospasm while environmental factors including cold environmental temperature, exertion and mental stress are relevant. The large international CLARIFY registry highlighted the importance of symptoms, showing that angina with or without concomitant ischaemia, was more predictive of adverse cardiac events compared with silent ischaemia alone.^15^ Other potential drivers of discordance between angina and ischaemia include variations in pain thresholds and cardiac innervation (eg, diabetic neuropathy).

### Symptoms and/or signs of ischaemia but no obstructive coronary artery disease (INOCA)

**Figure 2:**
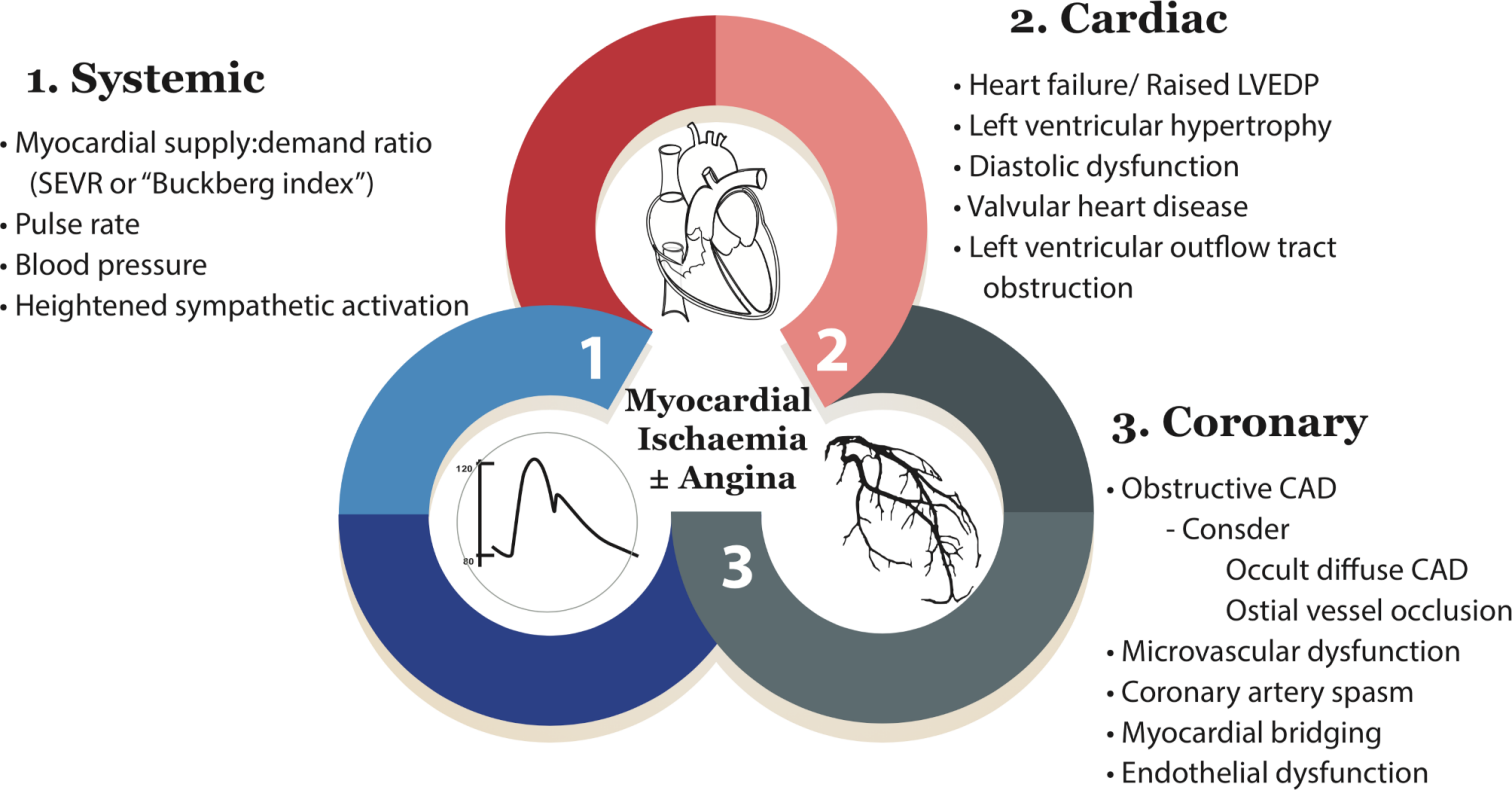
Contributing factors to myocardial ischaemia. The contributors to the physiological myocardial perfusion gradient and resultant ischaemia can be broken down at patient-level into systemic, cardiac and coronary factors. CAD, coronary artery disease; SEVR, subendocardial viability ratio.<Modified with permission from 47>.

Cardiologists are inclined to adopt a ‘stenosis centric’ approach to patient management; however, as clinicians we must appreciate that all factors are relevant, including coronary anatomy and function but systemic health and the psychosocial background (figure 2). First, **systemic** **factors** including heart rate, blood pressure (and their product) and myocardial supply:demand ratio (Buckberg index) are relevant.^16^ Reduced myocardial oxygen supply from problems such as anaemia or hypoxaemia should always be considered.

Second, **coronary** **factors** are well recognised but certain nuances are overlooked. In 2018, the first international consensus guidelines clarify that a definite diagnosis of MVA may be made in patients with angina with no underlying obstructive CAD, evidence of reversible ischaemia on functional testing and objective evidence of coronary microvascular dysfunction (table 1).^17^ ‘Probable MVA’ is defined by three of the above criteria. Coronary microvascular dysfunction may be structural (eg, small vessel rarefaction or increased media: lumen ratio) or functional (eg, endothelial impairment) and these disorders may coexist. Other coronary causes of INOCA include intramyocardial ‘tunnelled’ segments of epicardial arteries (myocardial ‘bridging’) who may have ischaemia on exercise. These segments are particularly susceptible to vasoconstriction due to endothelial impairment.^18^ Coronary arteriovenous malformations are rare but may also cause of myocardial ischaemia. Vasospastic angina (‘Prinzmetal’s angina’) is typically described as recurrent rest angina with focal occlusive proximal epicardial often seen in young smokers with characteristic episodic ST segment elevation during attacks. Notably, the more common form of VSA is distal and diffuse subtotal epicardial vasospasm and is characterised by ST segment depression and may occur during exertion. Typical cardiac risk factors and endothelial impairment may be implicated.^19^

The long-term (sometimes lifelong) burden of MVA and/or VSA on physical and mental well-being can be profound. Patients with these conditions commonly attend primary care and are repeatedly hospitalised with acute coronary syndromes, arrhythmias and heart failure driving up health resource utilisation, morbidity and reducing quality of life.^20 21^

The third and final group of factors that drive ischaemia in patients with angina but without obstructive CAD include **cardiac** **factors**. These include left ventricular hypertrophy or restrictive cardiomyopathy where subendocardial ischaemia results impaired perfusion from arterioles penetrating deeper into myocardial tissue with shorter diastole, enhanced systolic myocardial vessel constriction and enhanced interstitial matrix.^22^ Heart failure (with reduced or preserved ejection fraction) can lead to elevated left ventricular end diastolic pressure which reduces the diastolic myocardial perfusion gradient. Valvular heart disease (eg, aortic stenosis (AS)) is an important consideration in patients with INOCA. In AS, most experts support haemodynamic factors as the main cause of ischaemia, especially since symptoms and coronary flow reserve (CFR) improve immediately after valve replacement.^23^ Patients with INOCA may have increased painful sensitivity to innocuous cardiac stimuli (eg, radiographic contrast) without inducible ischaemia. Furthermore, some affected patients have a lower pain threshold and tolerance to the algogenic effects of adenosine (thought to be the main effector of ischaemia mediated chest pain).^24^

#### Gender differences and angina presentation

The WISE (Women’s Ischemia Syndrome Evaluation) study highlighted that over 2/3 of women with angina had no obstructive CAD and the majority of these had functional impairments in the coronary microcirculation associated with significant impairments in health-related quality of life.^25^ Indeed, women have more non-obstructive CAD and functional IHD which are frequently overlooked and hence undertreated.^26 27^ Over time and at different ages, women have a similar or slightly higher prevalence of angina than men across countries independent of diagnostic and treatment practices.^28^ Different patterns of IHD may be anticipated to cause different angina symptoms between genders. Nonetheless, recent evidence moves the field away from the ‘male-typical, female-atypical’ model of angina towards a ‘gender continuum’ whereby the objective reports between men and women’s symptoms are more similar than treating physicians perceive. Interestingly, dyspnoea was a feature in around ¾ of angina presentations without any significant difference between the sexes.^29^

## Assessment: current guidelines

Assessment strategies in current major international guidelines focus on the detection of underlying obstructive CAD. European and American guidelines (ESC and ACC/AHA, respectively) favour a Bayesian approach whereby overall probability of obstructive CAD after testing is determined from pretest probability modified by the diagnostic test results. The ACC/AHA guidelines determine pretest risk from a modified Diamond Forrester model,^30^ whereas the Europeans favour the CADC (Coronary Artery Disease Consortium) model which avoids overestimation seen with Diamond-Forrester and appears a more accurate assessment of pretest risk.^31^ Both current guidelines stratify pretest risk into low, intermediate or high groups with use of non-invasive testing suggested in the intermediate group (ACC/AHA arbitrarily defined as 10%–90% or 15%–85% in ESC).

In stark contrast, the NICE CG95 2016 update ‘chest pain of recent onset: assessment and diagnosis’ discarded the Bayesian pretest risk assessment. NICE advocates first-line multidetector CT coronary angiography (CTCA) in all patients with typical or atypical chest pain (box 1), those whose history does not suggest angina but who have ST changes or Q waves on a resting ECG.^32^ Functional testing (eg, exercise stress echo or stress perfusion magnetic resonance—CMR) are relegated to second-line if CTCA is non-diagnostic or the clinical significance of known CAD needs clarified. Potential benefits of this approach include a much higher diagnostic accuracy for detection of atherosclerotic heart disease than functional testing which potentially carries the best long-term prognostic information for patients with CAD.^27^ Extended 5-year outcomes from SCOTHEART showed a reduction in the combined endpoint of death from coronary heart disease or non-fatal myocardial infarction among the group randomised to CTCA compared with standard care (2.3% vs 3.9%; absolute risk reduction (ARR) 1.6% number needed to treat (NNT) 63). This effect was driven by better targeting of preventative therapies. The authors report that although overall prescriptions of preventive cardiovascular medications were only modestly increased (~10% higher) in the CTCA arm, changes in such therapies occurred in around one in four patients allowing more personalised treatment to patients with most coronary atheroma in the CT group.

These results should be considered in relation to design limitations of this trial. There was no control procedure (test vs no test), the threshold for prescribing preventive therapy with statins was 20%–30% likelihood of a CHD event in 10 years (much higher than many contemporary healthcare systems), CTCA was performed on top of treadmill exercise testing which has poor test accuracy in distinct patient groups, notably women, and the procedures were unblinded and open-label. Outcome reporting that is narrowly focused on CHD does not take account of other cardiovascular events, such as hospitalisation for arrhythmias and heart failure, which have implications for quality of life. In PROMISE, a ‘head-to-head’ trial of CTCA versus functional testing, there were no differences in health outcomes.^33^ In the interests of providing patients and clinicians with a reliable and accurate test result, a strategy based on anatomical CTCA has fundamental limitations. SCOT-HEART identified that obstructive CAD affects the minority (one in four) patients presenting to the Chest Pain Clinic with known or suspected angina. This means that an anatomical test strategy with CTCA leaving the aetiology and treatment unexplained in the majority of affected patients, which becomes all the more relevant considering that anginal symptoms and quality of life are worse when CTCA is used.^34^ Diagnostic options are enhanced by advances in technology and tests for the functional significance of CAD are now feasible, but at significant cost.^35^ NICE guidelines state that HeartFlow FFR_CT_ should be considered as an ‘option for patients with stable, recent onset chest pain who are offered CCTA as part of the NICE pathway on chest pain’. Using HeartFlow FFR_CT_ may avoid the need for invasive coronary angiography and revascularisation; however, major randomised controlled trials are ongoing (eg, FORECAST study NCT03187639).

We support efforts to provide a definitive diagnosis for patients with ongoing angina symptoms after a ‘negative’ CTCA, initially using non-invasive ischaemia testing. Notably, the recent International Standardised Criteria for diagnosing ‘suspected’ MVA would be met in patients with symptoms of myocardial ischaemia, no obstructive CAD and objective evidence of myocardial ischaemia (table 1). Invasive testing for diagnosis of MVA could be reserved for subjects with refractory symptoms and negative ischaemia testing or diagnostic uncertainty. The criteria for ‘definite MVA’ require the above AND objective evidence of microvascular dysfunction (eg, reduced CFR or raised microvascular resistance).

### Limitations of current guidelines

There are limitations to the current NICE-95 guideline, not least the logistics and cost of service provision with an estimated 700% increase in cardiac CT required across the UK.^36^ Importantly, what do we report to the majority of patients with anginal chest pain but no obstructive CAD on the CTCA? In fact, only 25% of patients had obstructive CAD and at 6 weeks based on the CTCA findings, 66% of patients were categorised as not having angina due to coronary heart disease. The possibility of false reassurance for the patients with angina and INOCA is an open question and may be one contributing factor for the lack of improvement in angina and quality of life in the CTCA group vs standard care.^34^ We must strive to deliver patient-centred care, recognising that most patients seek explanation for their symptoms in combination with effective treatment options.^37^ CTCA is an insensitive test for disorders of coronary vascular function, which may affect the majority of patients attending with anginal symptoms. Since the majority of affected patients have no obstructive CAD, and the majority of them are women, an anatomical strategy introduces a sex-bias into clinical practice, whereby a positive test result (obstructive CAD) is more likely to occur in men and a positive test for small vessel disease is less likely to occur in women. Furthermore, patient-reported outcomes including angina limitation, frequency and overall quality of life improve less after CTCA compared with standard care, notably in patients with no obstructive CAD.^34^ Non-invasive functional testing with positron emission tomography (PET), echo and most recently stress perfusion CMR has diagnostic value for stratified medicine. Finally, stratification of patients using luminal stenosis severity on angiography overlooks the spectrum of risk associated with overall plaque burden and may miss functional consequences associated with diffuse but angiographically mild disease (particularly when subtending large myocardial mass).

Non-invasive functional testing includes myocardial perfusion scintigraphy, exercise treadmill testing (including stress echocardiography) or contrast-enhanced stress perfusion MRI depending on local availability. Novel pixel-wise absolute perfusion quantification of myocardial perfusion by CMR will likely improve the efficiency of absolute quantification of myocardial blood flow by CMR.^38^ PET is the reference-standard non-invasive assessment of myocardial blood flow permitting quantitative flow derivation in mL/g/min. Clinically, PET-derived quantification of myocardial blood flow (MBF) can assist in the diagnosis of diffuse epicardial or microvascular disease; however, limitations include poor availability and exposure to ionising radiation. Non-invasive workup often provides important insights on coronary microvascular function and are reviewed in detail elsewhere.^39^

With functional testing relegated to second-line testing, clinicians may forgo additional tests after a negative CTCA particularly in an era of fiscal restraint and if patients’ symptoms are viewed as atypical. One important group that will be disparately affected by an ‘anatomy first’ strategy are women—over half of all patients with suspected angina in the large prospective trials of CTCA are female. While the benefits of CTCA to diagnose CHD and prevent CHD events are similar in women and men, the large majority of patients undergoing CTCA do not have obstructive CAD potentially leading to misdiagnosis and suboptimal management in patients with INOCA.^33^ Women, are most likely to have no obstructive CAD and their cardiac risk is associated with severely impaired CFR and not obstructive CAD.^40^ Overall, there is growing awareness of sex-specific differences in coronary pathophysiology and potential for different patterns of CAD in women. This is a rapidly evolving fertile area for further research.

### Invasive coronary angiography and physiological assessment

UK NICE guidelines suggest that invasive coronary angiography is a third-line investigation for angina when the results of non-invasive functional imaging are inconclusive. Patients with typical symptoms, particularly those in older age groups with higher probability of non-diagnostic CTCA scans, often proceed directly to invasive coronary angiography. During cardiac catheterisation, assuming that epicardial CAD is responsible for their symptoms, visual assessment for severe angiographic stenosis (>90%) is sufficient to establish significance and treatment plan for these patients. Two common pitfalls for visual interpretation of angiograms were recently highlighted by two coronary physiology pioneers Gould and Johnson. Using their quantitative myocardial perfusion database of over 5900 patients showing that occult coronary diffuse obstructive coronary disease or flush ostial stenosis may be both be overlooked on angiography and mislabelled as microvascular angina with suboptimal treatment.^41^ The ischaemic potential of indeterminate coronary lesions (~40%–70% diameter stenosis) is best assessed using pressure-derived indices, such as FFR, and non-hyperaemic pressure ratios (NHPR: dPR, nstantaenous wave free ratio (iwFR) and others) to guide revascularisation decisions. However, as is the case with coronary angiography, these indices do not inform the clinician about disorders of coronary artery vasomotion.

Invasive tests of coronary artery function are the reference standard for the diagnosis of coronary microvascular dysfunction^17^ and vasospastic angina (table 1; figure 1).^42^ Coronary microvascular resistance may be directly measured using guidewire-based physiological assessment during adenosine induced hyperaemia. Methods to assess this include using a pressure-temperature sensitive guidewire by thermodilution (index of microcirculatory resistance; IMR) or Doppler ‘ComboWire’ (hyperaemic microvascular resistance; HMR). These metrics have been the focus of a recent review article in Heart.^43^ There are several other haemodynamic indices of microvascular function including instantaneous hyperaemic diastolic pressure velocity slope, wave intensity analysis and zero flow pressure. A detailed description of these parameters is out with the scope of this review.^41^ Elevated coronary microvascular resistance (eg, IMR >25) carries prognostic utility in patients with reduced CFR but unobstructed arteries. Lee *et al* found over fivefold higher risk of adverse cardiac events in these subjects compared with controls with normal microvascular function.^44^

CFR is the ratio of maximum hyperaemic blood flow to resting flow. CFR in the absence of obstructive CAD can signify impaired microvascular dilation. Lance Gould first introduced this concept almost 50 years ago but more recently proposed that CFR should be considered in the context of the patient and the hyperaemic flow rate.^41^ The absolute threshold for abnormal CFR varies depending on the method of assessment, the patient population studies and the controversy reflects the dichotomous consideration of the continuous physiological spectrum of ischaemia. Abnormal CFR thresholds vary from ≤2.0 or ≤2.5 with more restrictive criteria for abnormal CFR (<1.6) being more specific for myocardial ischaemia and worse outcomes but at the cost of reduced sensitivity. On the other hand, studies of transthoracic Doppler derived CFR (which has less reproducibility) often use cut-offs of 2.5 with some observational evidence of worse outcomes in the INOCA population with CFR below this threshold.^45^ The influence of rate-pressure product on resting flow and its correction for CFR determination should be considered.

Systolic endocardial viability ratio (SEVR) is a ratio of myocardial oxygen supply:demand derived from the aortic pressure-time integral (diastole:systole). However, it is well known that blood pressure, pulse and SEVR perturbations influence CFR more closely than microcirculatory resistance. Reduced CFR without raised microvascular resistance still portends increased cardiovascular risk^44^ and may be a distinct subgroup with different drivers of ischaemia (eg, abnormal supply:demand systemic haemodynamic factors; figure 2). Alternatively, these patients may be at an earlier stage of disease prior to more established structural microvascular damage. Sezer *et al* showed the pattern of coronary microvascular dysfunction early in type II diabetes was driven by disturbed coronary regulation and high resting flow.^46^ In longstanding diabetes however, elevated microvascular resistance was observed reflecting established structural microvascular disease. This process matches the paradox of microvascular disease in diabetic nephropathy where increased glomerular filtration rate (GFR) typifies the early stages of disease prior to later structural damage and reduction in GFR.

The third mechanism of microvascular dysfunction is inappropriate propensity to vasoconstriction of the small coronary arteries, typically this is assessed using intracoronary acetylcholine infusions as a pharmacological probe.

#### Rationale and benefit of invasive coronary function testing in INOCA

We contend that a complete diagnostic evaluation of the coronary circulation should assess structural and functional pathology.^47^ The British Heart Foundation CorMicA trial provides evidence about the opportunity to provide a specific diagnosis to patients with angina using an interventional diagnostic procedure (IDP) when obstructive CAD is excluded by invasive coronary angiography. Consenting patients were randomised 1:1 to the intervention group (stratified medical therapy, IDP disclosed) or the control group (standard care, IDP sham procedure, results not disclosed). The diagnostic intervention included pressure guidewire-based assessment of FFR, CFR and IMR during adenosine induced hyperaemia (140 µg/kg/min). Vasoreactivity testing was performed by infusing incremental concentrations of acetylcholine (ACh) followed by a bolus vasospasm provocation (up to 100 µg). The diagnosis of a clinical endotype (microvascular angina, vasospastic angina, both, none) was linked to guideline-based management. The primary endpoint was the mean difference in angina severity at 6 months (as assessed by the Seattle Angina Questionnaire summary score—SAQSS) which was analysed using a regression model incorporating baseline score. A total of 391 patients were enrolled between 25/11/2016 and 11/12/2017. Coronary angiography revealed obstructive disease in 206 (53.7%). One hundred and fifty-one (39%) patients without angiographically obstructive CAD were randomised. The underlying abnormalities revealed by the IDP included: isolated microvascular angina in 78 (51.7%), isolated vasospastic angina in 25 (16.6%), mixed (both) in 31 (20.5%) and non-cardiac chest pain in 17 (11.3%). The intervention was associated with a mean improvement of 11.7 units in the SAQSS at 6 months (95% CI 5.0 to 18.4; p=0.001). In addition, the intervention led to improvements in the quality of life (EQ5D index 0.10 units; 0.01 to 0.18; p=0.024). After disclosure of the IDP result, over half of treating clinicians changed their diagnosis about the aetiology of their patients’ symptoms. There were no procedural serious adverse events and no differences in major adverse cardiac events (MACE) at 6 months. Interestingly, there were sustained quality of life benefits at one year for INOCA patients helped by correct diagnosis and linked treatment started at the index invasive procedure.^48^ Future trials are anticipated to determine the wider external validity of this approach.

## Management

Medical therapy to prevent new vascular events should be considered and these include consideration of aspirin, ACE inhibitors (ACEi) and statins. The latter two agents have pleiotropic properties including beneficial effects on endothelial function and so may be helpful in treating coronary microvascular dysfunction. Sublingual glyceryl trinitrate tablets or spray should be used for the immediate relief of angina and before performing activities known to bring on angina.

### Non-pharmacological

As with many cardiovascular diseases, lifestyle modification including risk factor control and patient education are key. Lifestyle recommendations are covered in detail in recent ESC guidelines. The adverse effect of angina on patient well-being and quality of life can be substantial. It is crucial that we assess for this and manage appropriately. After diagnosis with angina, cardiac rehabilitation can be useful to educate and build confidence. One useful patient led education aid is called the ‘Angina plan’. This tool is a workbook and relaxation plan delivered in primary care, which helps improve angina symptoms (frequency and limitation) while reducing anxiety and depression.^49^ The ORBITA trial highlights the benefits of placebo effect and we support that the positive diagnosis may be therapeutic in itself. Angina symptoms are often subjective and multifactorial in origin, so patient education and validation of symptoms may facilitate further improvement.

### Management: Non-obstructive CAD

Generic guidelines on angina management frequently overlooks the precision medicine goal whereby treatment is targeted to underlying pathophysiology. There is a lack of high-quality clinical trial data for treating microcirculatory dysfunction. The current article thus proposes a reasoned approach to management based on evaluation of pathophysiological mechanisms.

We contest that angina and INOCA are syndromes and not a precise diagnosis (akin to myocardial infarction with no obstructive CAD—MINOCA). As such, by stratifying treatment according to underlying pathophysiology, we may realise better outcomes for our patients.

#### Impaired coronary vasodilator capacity (reduced CFR)

Bairey Merz *et al* performed a randomised controlled trial of ranolazine in the WISE population. Notably, there was no net benefit effect on the INOCA population as a whole; however, in patients with reduced CFR (<2.5), there was a benefit suggestion of improved myocardial perfusion reserve index (MPRi) after established treatment.^50^ Lanza and Crea highlight that subjects with reduced CFR might preferentially be treated with drugs that reduce myocardial oxygen consumption (eg, beta-blockers (BB)—for example, Nebivolol 1.25–10 mg daily).^51^ There is accumulating evidence that long acting nitrates are ineffective or even detrimental in MVA. Lack of efficacy may relate to poor tolerability, steal syndromes through regions of adequately perfused myocardium and/or related to the reduced responsiveness of nitrates within the coronary microcirculation.^52^ Furthermore, chronic therapy with nitrate may induce endothelial dysfunction and oxidative stress, predominantly via endothelin dependent pathways.^53^

#### Increased microvascular constriction (structurally increased microvascular resistance or functional propensity to microvascular spasm)

Subjects with increased microvascular vasoconstriction may be treated with vasodilator therapies acting on the microcirculation. These include calcium channel blockers (CCB—for example, amlodipine 2.5–10 mg daily) or nicorandil (eg, 5–30 mg two times a day). Hyper-reactivity to constrictor stimuli resulting in propensity to microvascular spasm may be provoked by endothelial dysfunction. This was first described my Mohri *et al* over three decades ago with recent physiological studies suggesting treatment aimed at improving endothelial function (eg, ACEi, Ramipril 2.5–10 mg) may improve the microvascular tone and/or the susceptibility to inappropriate spasm.^54 55^ A detailed discussion of all potentially therapeutic options for coronary microvascular dysfunction is beyond the scope of this article; however, a systematic review by Marinescu *et al* may be of interest to readers wishing further information.^56^

#### Epicardial spasm (vasospastic angina)

The poor nitrate response or tolerance seen in MVA contrasts with patients with vasospastic angina, in whom nitrates are a cornerstone of therapy and BB are relatively contraindicated.^7^ Dual pathologies (VSA with underlying microvascular disease) is increasingly recognised. A diagnosis of VSA facilitates treatment using non-dihydropiridine calcium antagonists (eg, diltiazem-controlled release up to 500 mg daily). Overall, CCB are effective in treating over 90% of patients.^57^ High doses of calcium antagonists (non-dihydropiridine and dihydropyridine) may be required either alone or in combination. Unfortunately, ankle swelling, constipation and other side effects may render some patients intolerant. In these cases, long-term nitrates may be used with good efficacy in this group. In about 10% of cases, coronary artery spasm may be refractory to optimal vasodilator therapy. Japanese VSA registry data shows nitrates were not associated with MACE reduction in VSA, and importantly when added to Nicorandil were potentially associated with higher rates of adverse cardiac events.^58^ Alpha blockers (eg, clonidine) may be helpful in selected patients with persistent vasospasm. In patients with poor nitrate tolerance the K+-channel opener nicorandil (5–10 mg two times a day) can be tried. Consider secondary causes in refractory VSA (eg, coronary vasculitis) and in selected patients with ACS presentations, coronary angioplasty may be considered as a bailout option.

### Management: Obstructive CAD

#### Pharmacological

**Figure 3:**
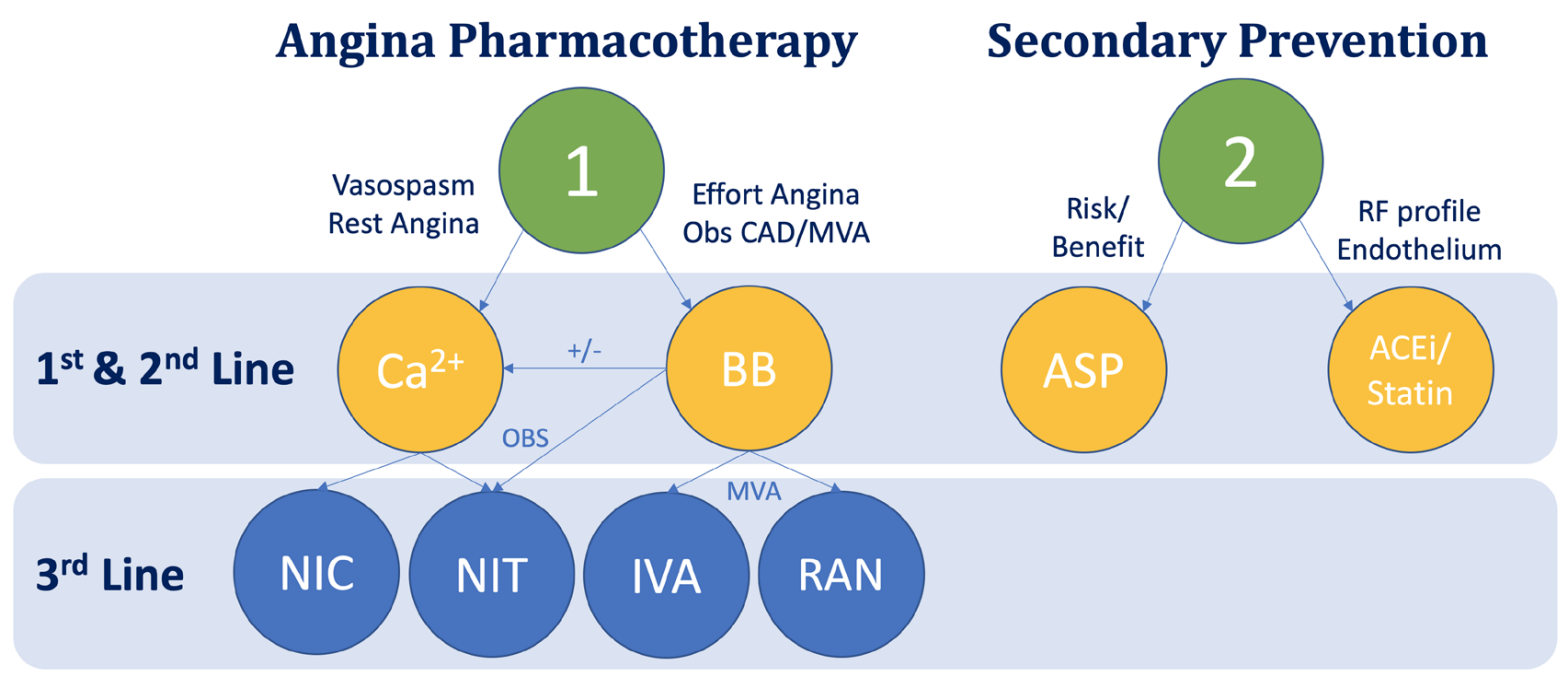
Empirical pharmacological treatments for patients with angina. ACEi, Angiotensin converting enzyme inhibitor; ASP, aspirin; BB, beta-blocker; Endo, endothelial; IVA, ivabradine; MVA, microvascular angina; NIC, nicorandil; NIT, nitrate; Obs CAD, obstructive coronary artery disease;, RAN, ranolazine; RF, risk factor.

Although NICE guidelines offer either BB or CCB first line, although we support BB initially because they are generally better tolerated (table 2).^59^ Long-term evidence of efficacy is limited between BB and CCB and there are no proven safety concerns favouring one or the other. Dihydropyridine calcium may be added to BB if blood pressure permits. NICE CG126 states third line options can be either added on (or substituted if BB/CCB not tolerated). These include nitrates (eg, isosorbide mononitrate 30–120 mg controlled release), ivabradine (eg, 2.5–7.5 mg two times a day), nicorandil (5–30 mg two times a day) or ranolazine (375–500 mg two times a day). These are all third line medications that can be used based and combined with BB and/or CCB depending on comorbidities, contraindications, patient preference and drug costs (figure 3). The RIVER-PCI study found that anti-ischaemic pharmacotherapy with ranolazine did not improve the prognosis of patients with incomplete revascularisation after percutaneous coronary intervention.^60^ This was a reminder that alleviation of ischaemia may not improve ‘hard’ endpoints in patients with chronic coronary syndromes but helps us to remain focused on improving their quality of life.

**Table 2:** Angina pharmacotherapy

Treatment	Angina type	Example	Investigation	Mechanism of action	Common side-effects
ß-blockers	MVA, CAD	Bisoprolol: 1.25–10 mg	Reduced CFR and/or structural microvascular dysfunction (raised microvascular resistance)	Reduction in myocardial oxygen consumption	Fatigue, blurred vision, cold hands
Calcium channel antagonists	All	Dihydropyridine (amlodipine: 2.5–10 mg daily) Non-dihydropyridine (verapamil: 40–240 or diltiazem up to 500 mg; controlled release)	Propensity to coronary vasospasm (epicardial and/or microvascular)	↓ spontaneous and inducible coronary spasm via vascular smooth muscle relaxation and ↓ oxygen demand Vascular smooth muscle relaxation, reduction in myocardial oxygen consumption	Constipation, ankle swelling, flushing
Vasodilators					
Nitrates	CAD, VSA	Isosorbid mononitrate: 30–120 mg one time a day (controlled released)	Propensity to epicardial coronary vasospasm	↓ spontaneous and inducible coronary spasm via large epicardial vasodilation, ↓ oxygen demand. Lack of efficacy in microvascular angina with potential deleterious effect	Headaches, dizziness, flushing
Nicorandil	All	Nicorandil: 5–30 mg two times a day	All	Potassium channel activator with coronary microvascular dilatory effect	Dizziness, flushing, weakness, nausea
Rho kinase inhibitors	VSA, CMD	Fasudil: 5–20 mg; three times a day	Epicardial and/or microvascular vasospasm	Reduce calcium sensitisation of vascular smooth muscle, maintains coronary vasodilation	Rashes, flushing, hypotension
Late Na+Current Inhibitors	MVA, CAD	Ranolazine: 375–500 mg two times a day	Reduced CFR	Improves MPRi in patients with MVA and reduced CFR	Nausea, dizziness, headache
I_f_ channel blockers	CAD, MVA	Ivabradine: 2.5–7.5 mg two times a day	All	Ivabradine has shown anti-ischaemic and antianginal activity	Bradycardia, AF, headache
Partial fatty-acid oxidation inhibitors	CAD, MVA	Perhexiline: 50–400 mg daily or Trimetazidine	Plasma concentration required for dose titration.	Perhexiline Inhibits carnitine O-palmitoyltransferase 1 and 2, which transfer free fatty acid from the cytosol into mitochondria.	Dizziness, unsteady, nausea and vomiting
Improved endothelial function/pleiotropic					
ACE inhibitors	MVA, CAD	Ramipril: 2.5–10 mg daily	Hyper-reactivity to stimuli (eg, acetylcholine, exercise, stress)	Improve CFR, reduce workload, may improve small vessel remodelling. Improves endothelial vasomotor dysfunction	Cough, renal impairment, hyperkalaemia
Statins	All	Atorvastatin: 10–80 mg daily Rosuvastatin: 5–40 mg daily	All	Improved coronary endothelial function reduced vascular inflammation	Myalgia, headache, cramps
Hormone-replacement therapy*	MVA	Oestradiol: 1 mg daily	Angina in early menopause	Oestrogen therapy improves endothelial function short-term in CMD	↑ Risk of breast cancer, marginally ↑ risk of CVD
Tricyclic antidepressants (TCA)	MVA with abnormal pain processing	Amitriptyline: 5–10 mg nocte Imipramine: 10–200 mg daily	All	Counteracts enhanced nociception. Thought to exert an analgesic effect on the visceral component associated with cardiac pain.	Blurred vision, dry mouth, drowsiness, impaired coordination
Non-pharmacological	All	Smoking cessation, Exercise, cardiac rehabilitation, Mediterranean diet, cognitive behavioural therapy, weight loss, Yoga	Metabolic syndrome, endothelial dysfunction, cardiovascular risk factors, anxiety/depression		Adjunctive non-pharmacological interventions

*May be helpful in some postmenopausal women. More information on experimentary pharmacotherapy in refractory angina can be found in review by Henry *et al*.^62^

CAD, angina with obstructive coronary artery disease; MPRi, myocardial perfusion reserve index; MVA, microvascular angina; VSA, vasospastic angina.

#### Revascularisation

Recently revised 2018 ESC guidelines suggest that myocardial revascularisation is indicated to improve symptoms in haemodynamically significant coronary stenosis with insufficient response to optimised medical therapy. Patients’ wishes should be accounted for in relation to the intensity of antianginal therapy as PCI can offer patients with angina and obstructive CAD a reduced burden from polypharmacy. Angina persists or recurs in more than one in five patients following PCI and microvascular dysfunction may be relevant. Guidelines support consideration of revascularisation for prognosis in asymptomatic ischaemia in patients with large ischaemic burden (left main/proximal left anterior descending artery stenosis >50%) or two/three vessel disease in patients with presumed ischaemia cardiomyopathy (LVEF<35%).

Refractory angina is common in patients with complex CAD including those with previous coronary artery bypass grafting (CABG) and chronic total occlusions (CTOs). Over the last decade, vast strides in technique, training and tools have delivered major increases in the success of CTO PCI. These angina patients often have incomplete revascularisation with lesions or anatomy previously considered ‘unsuitable for intervention’ but now amenable to treatment by trained operators. A recent review article in Heart summarises non-pharmacological therapeutic approaches to patients with refractory angina including cognitive behavioural therapy (CBT), stellate ganglion nerve blockade, Transcutaneous Electrical Nerve Stimulation (TENS)/spinal cord stimulation and pain modulating antidepressants (eg, imipramine).^61^ Of note, coronary sinus reducers deployed using a transcatheter venous system have shown early promise in clinical studies.

### Future directions

Based on test accuracy, health and economic benefits, non-invasive and invasive functional tests should be considered a standard of care in patients with known or suspected angina, especially if obstructive CAD has been excluded by CT or invasive coronary angiography. Computational fluid dynamic modelling of the functional significance of CAD, notably with FFRct, is an emerging option and clinical trials, including FORECAST (ClinicalTrials.gov Identifier: NCT03187639) and PRECISE (NCT03702244), are ongoing. The use of computational modelling as a diagnostic tool in patients with microvascular angina or coronary vasomotion disorders remains to be determined.

Systemic vascular abnormalities were recently highlighted in patients with INOCA potentially supporting a therapeutic role for targeted vascular therapy, for example, using selective endothelin-A receptor antagonists.^19^ The MRC Framework for Stratified Medicine is applicable to patients with angina and we believe genetic testing with precision medicine holds future promise.

## Conclusion

The optimal management of patients with known or suspected angina begins with establishing the correct diagnosis. Around one half of angina patients have no obstructive coronary disease; many of these patients have microvascular and/or vasospastic angina. Non-invasive assessment with CTCA is a sensitive anatomical test for plaque which assists in initial treatment and risk stratification. Anatomical imaging has fundamental limitations to rule in or rule out coronary vasomotion disorders in patients with symptoms and/or signs of ischaemia but no obstructive CAD (INOCA). Women are disproportionately represented in this group with MVA and/or VSA, the two most common causes of diagnoses. A personalised approach to invasive diagnostic testing permits a diagnosis to be made (or excluded) during the patients’ index presentation. This approach helps stratify medical therapy leading to improved patient health and quality of life. Physician appraisal of ischaemic heart disease (IHD) should consider all pathophysiology relevant to symptoms, prognosis and treatment to improve health outcomes for our patients. More research is warranted, particularly to develop disease modifying therapy.

### ESC curriculum: stable CAD

- Precipitants of angina.
- Prognosis of chronic IHD.
- Clinical assessment of known or suspected chronic IHD.
- Indications for, and information derived from, diagnostic procedures including ECG, stress test in its different modalities (with or without imaging, exercise and stress drugs) and coronary angiography.
- Management of chronic IHD, including lifestyle measures and pharmacological management.
- Indications for coronary revascularisation including PCI/stenting and CABG.

**Key points**

- Angina pectoris is a clinical syndrome occurring in patients with or without obstructive epicardial coronary artery disease.
- Diagnostic testing in angina is symptom driven and so should provide patients and their physicians with an explanation for their symptoms and used to stratify management and offer prognostic insights.
- Microvascular and/or vasospastic angina are common disorders of coronary artery function that may be overlooked by anatomical coronary testing, leading to false reassurance and adverse prognostic implications.

**CME credits for Education in Heart**

Education in Heart articles are accredited for CME by various providers. To answer the accompanying multiple choice questions (MCQs) and obtain your credits, click on the 'Take the Test' link on the online version of the article. The MCQs are hosted on BMJ Learning. All users must complete a one-time registration on BMJ Learning and subsequently log in on every visit using their username and password to access modules and their CME record. Accreditation is only valid for 2 years from the date of publication. Printable CME certificates are available to users that achieve the minimum pass mark.

10.1136/heartjnl-2018-314661.supp1 Supplementary data
