# Supplementary material for: Angina: contemporary diagnosis and management
Source: Heart. 2020 Feb 12;106(5):387–98. doi: 10.1136/heartjnl-2018-314661 (PMC7035719; doi:10.1136/heartjnl-2018-314661)
Supplement: Supplementary data [file heartjnl-2018-314661supp001.pdf]

MCQs

Angina: Contemporary Diagnosis and Management

Authors: Thomas J Ford [1,2], Colin Berry [1,2]

Institutions: [1] West of Scotland Heart and Lung Centre, Golden Jubilee National Hospital, UK; [2] British Heart Foundation Glasgow Cardiovascular Research Centre, Institute of Cardiovascular and Medical Sciences, University of Glasgow, UK;

Q1. Which of the following statements best describes coronary flow reserve (CFR):

A. CFR is a specific quantitative metric to assess coronary microvascular function

B. CFR reflects endothelial dependent coronary vasorelaxation

C. CFR is a ratio of maximal achievable myocardial blood flow to resting blood flow

D. CFR is a more reproducible test of coronary function compared to fractional flow reserve (FFR)

E. None of the above

Answer: C

CFR can be thought of as the capacity of the coronary circulation to dilate and thus increase flow following an increase in myocardial metabolic demands. CFR is inherently variable and less reproducible than FFR due to its association with resting haemodynamics. Invasive assessment of CFR is typically performed before and after adenosine induced hyperaemia – it predominantly reflects endothelial independent function.

Q2: Which of the following best describes typical stable angina

A. Chest discomfort occurring at rest with a fixed duration

B. Recent onset angina occurring with a fixed amount of exertion relieved with rest

C. Retrosternal chest discomfort occurring with a fixed amount of exertion rapidly relieved with rest or GTN

D. Angina occurring with a fixed amount of exertion that has recently required less exertion to bring about symptoms

E. Exertional presyncope and dyspnoea

Answer: C

Diamond criteria for typical (definite) angina has three components[1]:

1. Substernal chest discomfort with a characteristic quality and duration
2. Provoked by exertion or emotional stress
3. Relieved by rest or nitro-glycerine

These features of angina are incorporated into the ESC guidelines on management of stable angina.[2]

Q3: Which of the following investigations is most sensitive for the diagnosis of coronary artery plaque

A. Coronary angiography
B. Exercise stress ECG
C. Exercise stress echo
D. CT coronary angiography
E. Stress perfusion magnetic resonance imaging

Answer D

CT coronary angiography is the most sensitive tool for the diagnosis epicardial coronary artery plaque.[3] In correlation study with histology, the diagnostic accuracy of CT to detect

calcified plaque was 83%.[4] Invasive angiography may miss epicardial plaque without luminal obstruction – this is frequently due to Glagov's phenomenon whereby positive remodelling without lumen encroachment occurs until approximately 50% plaque burden by cross sectional area. Functional testing is more specific for the ischaemic potential of epicardial coronary artery disease but is insensitive for the diagnosis of coronary artery plaque.

Q4: 54-year-old female with angina and abnormal stress ECG undergoes coronary angiography and is found to have non obstructive coronary artery disease. Which of the following is true?

A. Cardiovascular risk is similar to an asymptomatic age/sex matched control
B. Angina pectoris is excluded
C. Preventative cardiovascular medicines should be stopped (statin)
D. Coronary vasomotion disorder should be considered
E. Antianginal therapy should cease

Answer D

This scenario of a patient with symptoms and/or signs of ischaemia and no obstructive coronary artery disease (INOCA) is increasingly recognised. Diffuse but non obstructive plaque disease is associated with an increased cardiovascular risk which is more pronounced in women more than men.[5] Coronary vasomotion disorders should be considered as a unifying diagnosis for this lady with primary microvascular angina the most common cause of INOCA. She should be treated with beta-blockers in the first instance.[6]

Q5: Which of the following meets diagnostic criteria for definite vasospastic angina?

A. Nitrate responsive angina without obstructive coronary artery disease
B. Angina with diurnal variation, transient ischaemia on ECG monitoring and transient total or subtotal coronary artery occlusion (>90% constriction) with provocation during angiography
C. Atypical chest pain, negative stress ECG but T wave inversion and 50% LAD constriction with ACh during coronary angiography
D. Unexplained cardiac arrest preceded by chest pain with irregular non obstructive lesion on coronary angiogram
E. Atypical chest pain and dyspnoea during mental stress

Answer B

The first international standardised guidelines on diagnosis of VSA state: ‘Definitive vasospastic angina’ is diagnosed if nitrate-responsive angina is evident during spontaneous episodes and either the transient ischaemic ECG changes during the spontaneous episodes or coronary artery spasm criteria are fulfilled. Spasm criteria require >90% constriction of epicardial coronary artery spontaneously or in response to acetylcholine with reproduction of angina and ischaemic ECG changes”.[7] Calcium channel blockers are very effective first line therapy in over 90% of cases.

Q6: Which of the following statements regarding myocardial revascularisation is correct?

A. Improves prognosis for certain patient groups or specific subsets of coronary anatomy

B. Has no proven benefit unless CAD involves the left main coronary artery
C. Visual assessment of the coronary angiogram is the gold standard for determining whether revascularisation of a coronary stenosis is appropriate
D. Patients should be on at least three concurrent antianginal agents before considering revascularisation
E. All patients with coronary artery disease should be discussed at a heart team (multidisciplinary meeting) before undergoing myocardial revascularisation.

Answer A.

Recent evidence shows that compared to medical therapy alone, CAD patients randomised to coronary revascularisation with either stents[8] or coronary artery bypass grafting (CABG)[9] have more effective angina reduction and lower risk of major adverse cardiac events. The ESC guidelines on management of stable coronary artery disease support myocardial revascularisation to improve symptoms in haemodynamically significant coronary stenosis with insufficient response to optimized medical therapy. Patient wishes should be taken into account regarding the intensity of anti-anginal therapy. Revascularisation for asymptomatic ischaemia may be considered in patients with large ischaemic burden (left main/proximal left anterior descending artery stenosis >50%) or two/three vessel disease in patients with presumed ischaemia cardiomyopathy (LVEF <35%).[2] The visual assessment of a coronary angiogram may be misleading and invasive physiological interrogation of a stenosis may help to determine the ischaemic potential of a lesion. Not all patients with CAD need discussed at heart team meetings (e.g. single vessel disease with simple anatomy).

References

1. Diamond GA. A clinically relevant classification of chest discomfort. *J Am Coll Cardiol* 1983;1(2 Pt 1):574-5. [published Online First: 1983/02/01]

2. Task Force M, Montalescot G, Sechtem U, et al. 2013 ESC guidelines on the management of stable coronary artery disease: the Task Force on the management of stable coronary artery disease of the European Society of Cardiology. *Eur Heart J* 2013;34(38):2949-3003. doi: 10.1093/eurheartj/eht296

3. Stefanadis C, Antoniou CK, Tsiachris D, et al. Coronary Atherosclerotic Vulnerable Plaque: Current Perspectives. *J Am Heart Assoc* 2017;6(3) doi: 10.1161/JAHA.117.005543

4. Obaid DR, Calvert PA, Gopalan D, et al. Atherosclerotic plaque composition and classification identified by coronary computed tomography: assessment of computed tomography-generated plaque maps compared with virtual histology intravascular ultrasound and histology. *Circ Cardiovasc Imaging* 2013;6(5):655-64. doi: 10.1161/circimaging.112.000250 [published Online First: 2013/08/21]

5. Sedlak TL, Lee M, Izadnegahdar M, et al. Sex differences in clinical outcomes in patients with stable angina and no obstructive coronary artery disease. *Am Heart J* 2013;166(1):38-44. doi: 10.1016/j.ahj.2013.03.015

6. Ford TJ, Corcoran D, Oldroyd KG, et al. Rationale and design of the British Heart Foundation (BHF) Coronary Microvascular Angina (CorMicA) stratified medicine clinical trial. *Am Heart J* 2018;201:86-94. doi: 10.1016/j.ahj.2018.03.010 [published Online First: 2018/05/29]

7. Beltrame JF, Crea F, Kaski JC, et al. International standardization of diagnostic criteria for vasospastic angina. *Eur Heart J* 2017;38(33):2565-68. doi: 10.1093/eurheartj/ehv351 [published Online First: 2015/08/08]

8. Zimmermann FM, Omerovic E, Fournier S, et al. Fractional flow reserve-guided percutaneous coronary intervention vs. medical therapy for patients with stable coronary lesions: meta-analysis of individual patient data. *Eur Heart J* 2019;40(2):180-86. doi: 10.1093/eurheartj/ehy812 [published Online First: 2019/01/01]

9. Windecker S, Stortecky S, Stefanini GG, et al. Revascularisation versus medical treatment in patients with stable coronary artery disease: network meta-analysis. *BMJ* 2014;348:g3859. doi: 10.1136/bmj.g3859 [published Online First: 2014/06/25]
